# Metastatic Prostate Cancer Masquerading as Locally Advanced Breast Cancer

**DOI:** 10.1002/ccr3.72452

**Published:** 2026-04-06

**Authors:** Tess Howard, Mylestone Gobe Monna, Jasmine Zhu, Jiasian Teh, Sze Ting Lee, Grace L Chew

**Affiliations:** ^1^ Department of General Surgery Austin Health Melbourne Australia; ^2^ Department of Anatomical Pathology Austin Health Melbourne Australia; ^3^ Department of Urology Austin Health Melbourne Australia; ^4^ Department of Nuclear Medicine Austin Health Melbourne Australia; ^5^ Division of Surgery Northern Health Epping Australia

**Keywords:** breast neoplasms secondary, gynecomastia, positron emission tomography, prostatic neoplasms

## Abstract

We report a rare case of a 72‐year‐old man with prostatic adenocarcinoma metastasizing to gynecomastia breast tissue with ipsilateral axillary lymph node involvement. This presentation is notable for the absence of disseminated metastatic disease or prolonged androgen deprivation therapy. The clinical and radiological features initially suggested a primary breast carcinoma, highlighting the diagnostic challenge in such presentations. The patient had a history of high‐risk prostate adenocarcinoma resected in 2020. A prostate‐specific membrane antigen (PSMA) PET scan, performed for rising prostate‐specific antigen (PSA) levels, demonstrated a new avid left breast lesion within longstanding bilateral gynecomastia tissue. The pattern of PSMA uptake, including involvement of ipsilateral axillary lymph nodes, further increased suspicion for a locally advanced primary breast carcinoma. Core biopsy of the breast lesion and fine‐needle aspiration of the axillary lymph node were consistent with metastatic prostate adenocarcinoma, which was subsequently confirmed on surgical excision. This case adds to the limited literature describing prostate cancer metastasis to the breast and underscores the diagnostic challenges such presentations can create. It demonstrates how metastatic disease can closely mimic primary breast malignancy, reinforcing the importance of histopathological confirmation when imaging findings suggest a primary breast lesion.

## Key Clinical Message

Metastatic prostate carcinoma to the breast is rare and may resemble primary breast cancer, creating diagnostic uncertainty. Clinicians should consider metastatic disease when evaluating breast lesions in patients with a history of malignancy. Tumor type and clinical context should be considered, and surgery may aid diagnostic confirmation and disease control.

## 1 Introduction

Metastatic involvement of the breast from extramammary malignancies is exceedingly rare in both sexes, accounting for approximately from 0.3% to 2.7% of all breast tumors [1]. Common primary malignancies that metastasize to the breast include hematological cancers, melanoma, carcinomas of the lung and, in women, the ovary [2]. Male breast cancer is also uncommon, representing less than 1% of all cancers in men and less than 1% of all breast cancers [3].

Conversely, prostate adenocarcinoma is one of the most frequently diagnosed malignancies in men, with a predilection for metastasis to bones and lymph nodes [4]. Cases of prostate cancer metastasizing to the breast are notably rare, with the majority reported between the 1940s and 1980s, when estrogen therapy was the primary hormonal treatment for prostate cancer [5]. In more recent years, isolated cases of metastatic deposits to the breast have been described, most commonly in the context of advanced systemic disease and/or prolonged androgen deprivation therapy (ADT) [6].

Here, we present a case that is unusual in several features. The breast metastases occurred in the absence of disseminated metastatic disease, in a patient with a limited history of ADT, and were associated with ipsilateral axillary lymphatic involvement, closely mimicking the presentation of a locally advanced primary breast carcinoma.

## 2 Case Presentation

A 72‐year‐old man was initially diagnosed with prostatic adenocarcinoma in January 2020. This diagnosis was based on clinical and pathological findings, confirmed through imaging and a transperineal prostate biopsy. His initial staging PSMA PET scan showed no evidence of metastatic disease, and his initial PSA was 7 μg/L.

In February 2020, he underwent a robotic prostatectomy. The surgical specimen revealed prostate cancer classified as T3aN0M0 with extracapsular extension, Gleason Score of 4 + 5 = 9 and negative resection margins. Due to persistently elevated PSA levels, which were considered to represent biochemical recurrence, he was treated postoperatively with radiotherapy to the prostatic bed and pelvic nodal basins in addition to a short course of ADT consisting of two Goserelin 10.8 mg injections, 12 weeks apart. This resulted in complete biochemical remission.

His past medical history included benign prostatic hyperplasia managed for 18 months with dutasteride/tamsulosin prior to 2020. He also had longstanding hypertension treated with spironolactone. The prolonged spironolactone exposure likely contributed to the development of bilateral moderate gynecomastia, with prior dutasteride exposure and the short course of androgen deprivation therapy acting as additional contributors.

In March 2024, the patient's serum PSA rose to 0.30 μg/L, prompting repeat PSMA PET imaging. This demonstrated a single avid lesion in the left mesorectum, consistent with local nodal recurrence. Incidentally, the PSMA scan also revealed a new lumbar paraspinal soft tissue mass suspicious for a soft tissue sarcoma. Symmetrical PET uptake was also observed in bilateral gynecomastia breast tissue, without the presence of any distinct lesions or lymphadenopathy. In the following months, the patient underwent two courses of radiotherapy, including stereotactic radiation directed to the mesorectal node and neoadjuvant radiotherapy to the sarcoma site. The sarcoma was subsequently excised by a specialized sarcoma service in July 2024, with final histopathology confirming the presence of an undifferentiated pleomorphic sarcoma.

**FIGURE 1:**
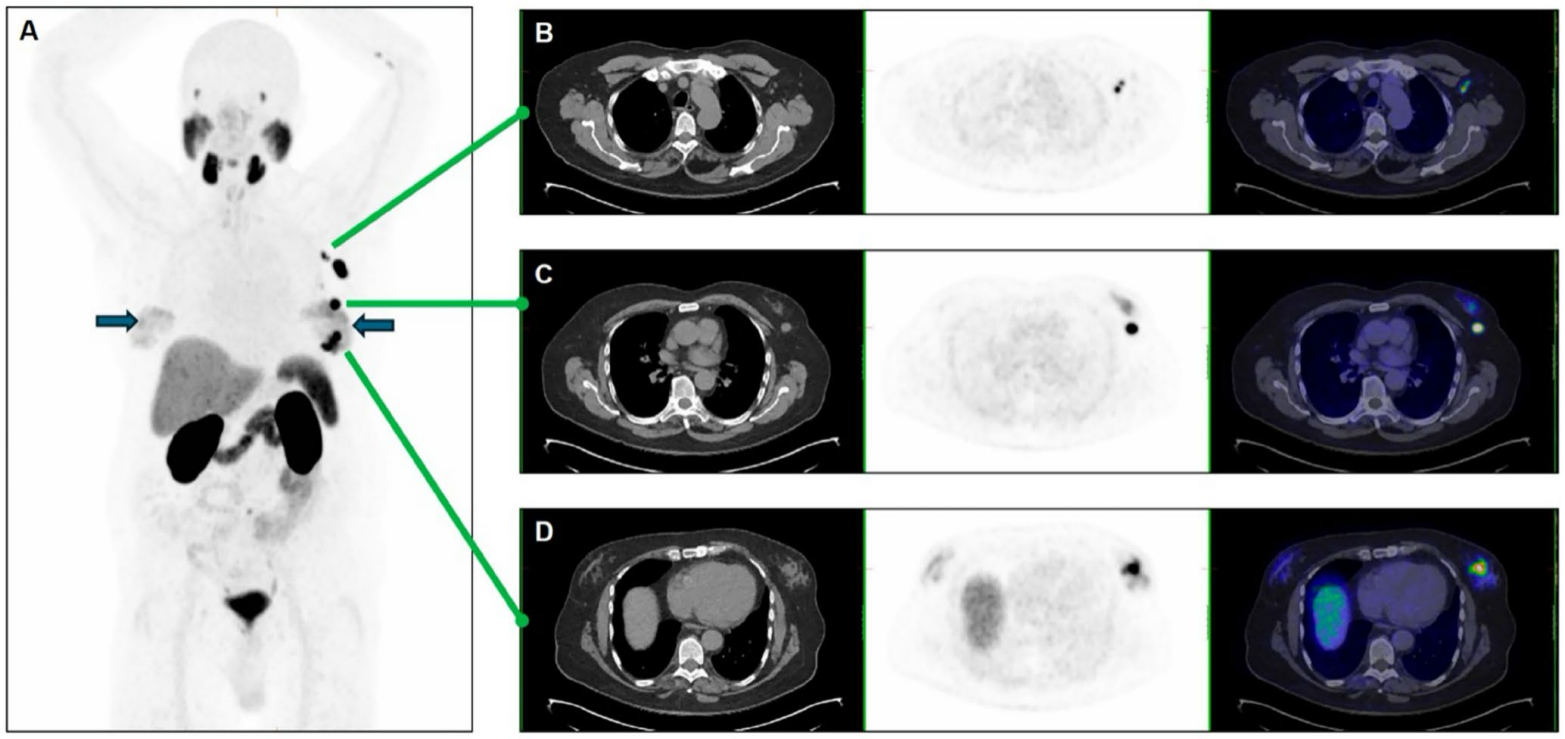
A: Whole body 68Ga‐PSMA PET/CT scan demonstrating multiple PSMA‐avid lesions in the left breast on background of mildly PSMA‐avid gynecomastia (solid arrows). Physiologic PSMA activity in the lacrimal glands, salivary glands, liver, spleen, kidneys, bowel, ureters, and urinary bladder. Panel B: Small PSMA‐avid left axillary nodes on CT, PET and fused images. Panels C & D: Multifocal PSMA‐avid left breast lesions on CT, PET and fused images. The combination of a focal breast lesion with ipsilateral axillary nodal uptake created an imaging pattern closely resembling a primary breast carcinoma with regional nodal drainage.

Despite these interventions, a further PSA rise was observed post‐radiotherapy, with levels peaking at 4.56 μg/L by October 2024. This prompted a repeat PSMA PET scan, which demonstrated asymmetric focal intense tracer uptake within the left gynecomastia tissue with corresponding ipsilateral axillary nodal avidity (Figure 1). The pattern of imaging findings, including asymmetric focal uptake within a discrete breast lesion, associated regional axillary nodal drainage, and interval progression on serial imaging in the absence of other new sites of disseminated disease, raised concern for a primary breast malignancy rather than metastatic disease. The patient was therefore referred to the breast surgery unit for further evaluation.

Physical examination revealed bilateral gynecomastia with a firm, mobile mass in the left 4 o'clock periareolar region, measuring 2 × 1.5 cm. A prominent, palpable left axillary lymph node was also identified. Ultrasound of the bilateral chest wall confirmed the presence of an ill‐defined, irregular hypoechoic mass in the left breast at the 4 o'clock position, immediately adjacent to the areola. It measured 27 × 14 × 15 mm and was categorized as BI‐RADS 4 B. Additionally, two left axillary lymph nodes demonstrated suspicious features, including abnormal cortical thickening up to 8 mm. These findings were deemed consistent with the PSMA PET results. On the contralateral side, ultrasound demonstrated gynecomastia without any suspicious lesions or lymphadenopathy.

Given the atypical presentation, further diagnostic evaluation was undertaken to exclude the possibility of primary breast malignancy. Although core biopsy of the breast lesion and fine‐needle aspiration of the axillary node were highly suggestive of metastatic prostate carcinoma, the radiological pattern of a discrete breast mass with ipsilateral axillary nodal involvement remained more typical of a primary breast malignancy.

The case was reviewed at both the breast and urology multidisciplinary meetings. Discussion focused on the discordance between the imaging findings and biopsy results, the unusual radiological pattern of a discrete breast lesion with regional nodal involvement without other sites of metastatic disease, and the oligometastatic nature of the presentation. In this context, surgical management was considered an appropriate strategy for both local disease control and definitive histopathological assessment. Following multidisciplinary consensus, the patient proceeded to simple mastectomy and axillary lymph node dissection in December 2024.

## 3 Histopathology

Macroscopic examination of the left mastectomy specimen demonstrated a tumor measuring 35 × 25 × 15 mm (ML × SI × AP) with a nodular tan cut surface and focal hemorrhagic areas.

**FIGURE 2:**
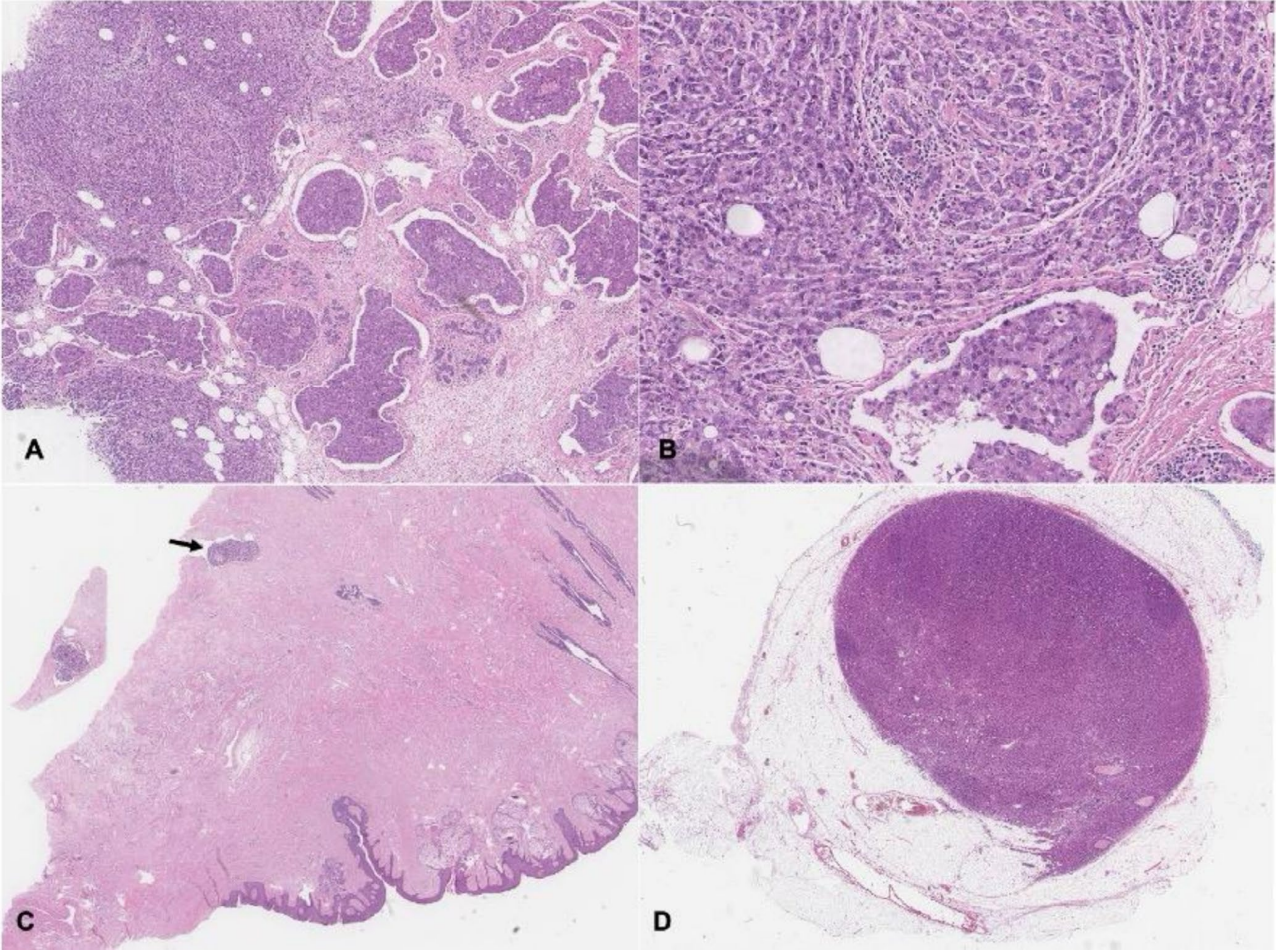
A. Low‐power scanning view showing tumor with nested and sheet‐like architecture. B. High‐power view of tumor cells demonstrating pleomorphic and hyperchromatic nuclei with eosinophilic cytoplasm. C. Lymphovascular invasion by tumor, deep to the nipple (arrow). D. Apical lymph node completely replaced by tumor with extranodal extension. These morphological features are not typical of primary breast carcinoma and raised suspicion for metastatic disease, prompting further immunohistochemical evaluation to determine tumor origin.

Histopathological examination showed features in keeping with metastatic prostate carcinoma (Figure 2). Sections demonstrated malignant cells infiltrating the breast parenchyma in sheets and large nests, characterized by large pleomorphic nuclei, coarse chromatin, prominent nucleoli, and variable amounts of eosinophilic cytoplasm. Round cytoplasmic vacuoles were frequently identified. Extensive lymphovascular invasion was present, including within tissue deep to the nipple, distant from the main tumor bulk. Surgical margins were clear. The background breast parenchyma showed features of gynecomastia, including variably expanded lobules with acini lined by mitotically active epithelial cells.

Sections from the left axillary dissection demonstrated metastatic carcinoma in six of thirteen lymph nodes (6/13). The apical node was entirely replaced by tumor, with extranodal extension and intravascular tumor identified in the surrounding tissue.

**FIGURE 3:**
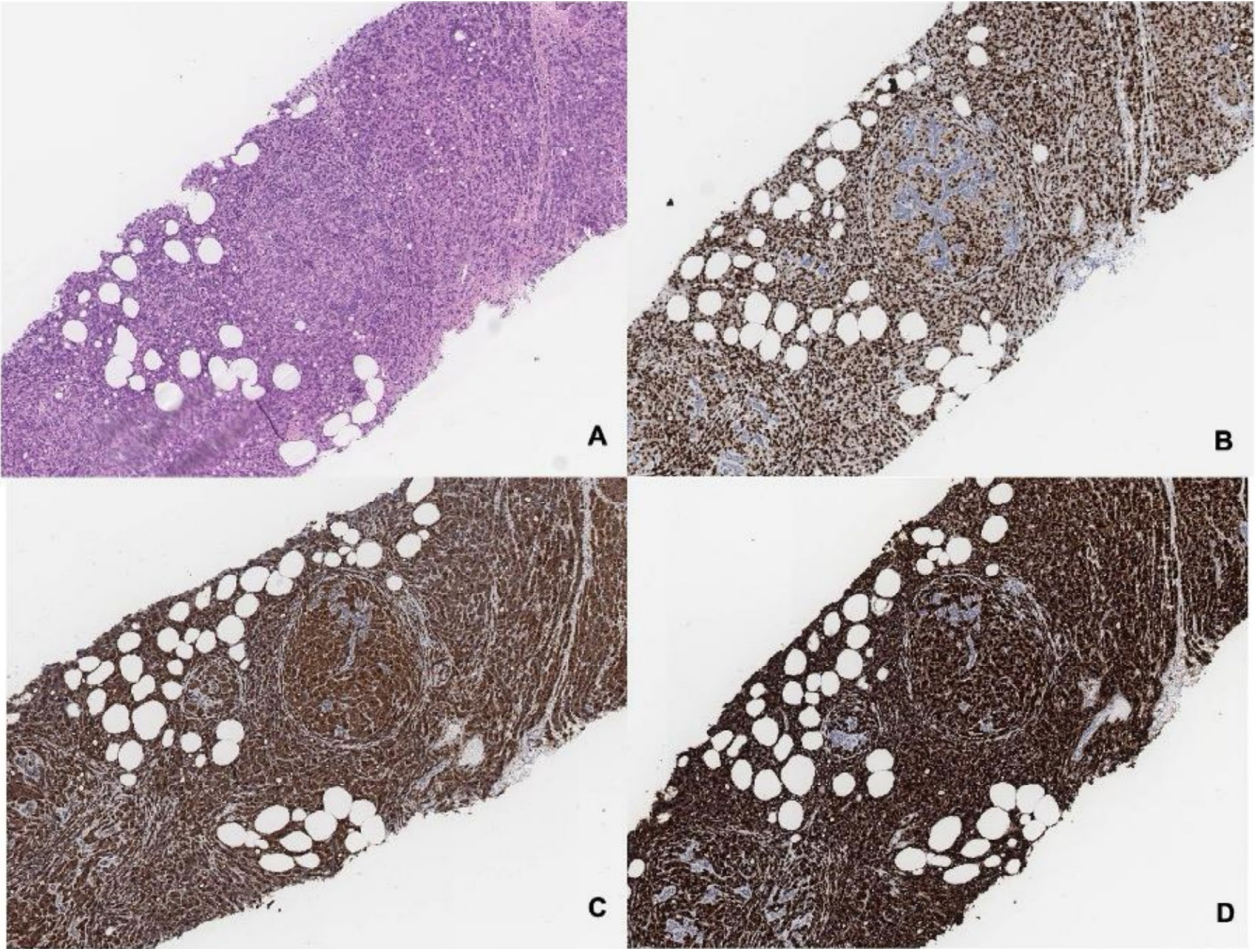
A. Left breast core biopsy showing tumor. B. Tumor cells showing positive NKX3.1 staining. C. Diffuse strong expression of PSA in tumor cells. D. Tumor cells showing diffuse strong expression of PSMA. The expression of prostate‐specific markers (NKX3.1, PSA, and PSMA) confirms prostatic origin of the tumor and supports the diagnosis of metastatic prostate adenocarcinoma rather than primary breast carcinoma.

Tumor morphology within the mastectomy specimen was similar to that seen in the previous core biopsies. Immunohistochemistry performed on the biopsies demonstrated tumor cells positive for NKX3.1, with diffuse strong expression of prostate‐specific antigen (PSA) and prostate‐specific membrane antigen (PSMA). GATA3 showed weak to moderate expression; however, GATA3 positivity can be seen in several non‐breast malignancies and therefore required correlation with prostate‐specific markers, including NKX3.1, PSA, and PSMA, supporting a prostatic origin. Tumor cells were negative for estrogen receptor (ER) (Figure 3).

## 4 Clinical Progress

The case was reviewed at both the breast and urology multidisciplinary meetings. Adjuvant radiotherapy to the chest wall and axilla was considered but not recommended given the clear surgical margins, absence of disseminated disease, and the planned systemic therapy. The patient was also referred for genetic testing, which demonstrated no abnormality on a 20‐gene hereditary cancer panel covering genes associated with breast, ovarian, pancreatic, and prostate cancer susceptibility.

Systemic therapy was commenced with Goserelin 10.8 mg subcutaneously every three months and Darolutamide 600 mg orally twice daily. At six‐month follow‐up, the PSA remains suppressed at 0.04 μg/L. Surveillance CT of the chest, abdomen and pelvis and MRI of the spine show no evidence of recurrent prostate carcinoma or sarcoma.

## 5 Discussion

Metastasis of prostate cancer to the breast is exceedingly rare. A greater number of cases were described in the 1970s and 1980s, when estrogen therapy was commonly used for prostate cancer; however, reports have become increasingly scarce since the discontinuation of this treatment. Although contemporary hormonal therapies such as androgen deprivation therapy (ADT) differ mechanistically, they can still alter the androgen–estrogen balance and have been associated with proliferative changes in breast tissue and the development of gynecomastia [7, 8].

In the present case, the patient had longstanding spironolactone‐associated gynecomastia and prior exposure to dutasteride, both of which may influence androgen–estrogen balance. The patient also received a short course of androgen deprivation therapy, although the duration of exposure was limited. We hypothesize that these combined hormonal influences may have altered the local breast microenvironment. Whether such endocrine changes could facilitate metastatic tumor implantation remains speculative.

Several recent case reports have described prostate carcinoma metastasizing to the breast, most commonly in the setting of advanced systemic disease or prolonged androgen deprivation therapy, with diagnosis typically established through biopsy alone [6, 9, 10]. In contrast, the present case occurred in the absence of disseminated metastatic disease and demonstrated a radiological pattern closely resembling a primary breast carcinoma with regional nodal involvement.

PSMA PET/CT has become an invaluable tool in the detection and staging of recurrent prostate cancer, demonstrating high sensitivity even at low PSA levels. However, PSMA expression is not prostate cancer‐specific and can be observed in a range of other malignancies, including breast, renal, and hepatocellular carcinomas, as well as various sarcomas. Uptake may also occur in benign conditions, such as gynecomastia, fractures, or inflammatory processes [11]. Increasingly, incidental detection of non‐prostate cancers on PSMA PET has been reported, which, while clinically valuable, may create diagnostic ambiguity. Importantly, the pattern of a focal breast lesion with ipsilateral nodal drainage strongly mirrors the expected presentation of a primary breast carcinoma and may therefore bias interpretation toward a primary tumor of that organ, even when the imaging tracer itself is not disease specific. In this case, the pattern of avidity in the left breast and ipsilateral axilla was more consistent with a primary breast carcinoma, underscoring the need for histopathological confirmation prior to definitive treatment.

The development of extramammary metastases is generally considered a poor prognostic feature and typically reflects advanced disease [2]. Breast involvement from prostate cancer is exceptionally rare, and there are no established treatment guidelines; management is therefore usually extrapolated from principles used in metastatic prostate cancer more broadly [12]. In this case, surgical resection was undertaken following multidisciplinary discussion primarily to achieve local disease control in the setting of isolated oligometastatic recurrence, as part of an integrated strategy with systemic therapy. Although core biopsy findings were strongly supportive of metastatic prostate carcinoma, the discrepancy between the radiological pattern, clinical presentation, and histopathology left residual diagnostic uncertainty. Excision therefore provided both definitive local control and confirmatory tissue diagnosis. Importantly, the morbidity associated with simple mastectomy and axillary lymph node dissection is relatively low, supporting the appropriateness of this individualized approach.

This case reinforces several key clinical principles: the importance of considering metastasis in new breast lesions in men with a prior cancer history, the limitations of imaging alone in differentiating primary from secondary tumors, and the critical role of histopathology and immunohistochemistry in guiding diagnosis and treatment. In particular, prostate‐specific markers such as PSA, PSMA, and NKX3.1 provide decisive evidence of tumor origin and may resolve diagnostic uncertainty created by imaging findings and clinical context.

As outcomes for prostate cancer continue to improve with advances in systemic therapy, clinicians may encounter less typical patterns of recurrence. New or unusual lesions in patients with a prior history of prostate malignancy should therefore be assessed carefully, with consideration of metastatic disease even when the site or presentation is atypical.

## 6 Conclusion

This case highlights an exceptionally rare presentation of metastatic prostate adenocarcinoma involving the breast with ipsilateral axillary lymph node involvement, closely mimicking locally advanced primary breast cancer. It underscores the limitations of imaging pattern recognition alone in distinguishing primary breast malignancy from metastatic disease, even in the era of highly sensitive modalities such as PSMA PET. In this case, the presence of an isolated oligometastatic lesion with regional nodal involvement further contributed to the diagnostic ambiguity. Accurate diagnosis therefore relies on careful integration of clinical context, imaging findings, and definitive histopathological confirmation through immunohistochemistry.

Awareness of rare metastatic presentations such as breast involvement is important to avoid misdiagnosis and inappropriate management. Multidisciplinary evaluation remains critical to ensure that treatment strategies are appropriately tailored for these uncommon and diagnostically challenging presentations.

## Author Contributions

**Tess Howard:** conceptualization, writing – original draft, writing – review and editing. **Mylestone Gobe Monna:** writing – original draft, writing – review and editing. **Jasmine Zhu:** writing – original draft, writing – review and editing. **Jiasian Teh:** writing – review and editing. **Sze Ting Lee:** writing – review and editing. **Grace L Chew:** conceptualization, resources, supervision, writing – review and editing.

## Funding

The authors have nothing to report.

## Consent

Written informed consent was obtained from the patient to publish this report in accordance with the journal's patient consent policy.

## Conflicts of Interest

The authors declare no conflicts of interest.

## Data Availability

Data sharing not applicable to this article as no datasets were generated or analysed during the current study.
